# Supplementary material for: Group V Chitin Deacetylases Influence the Structure and Composition of the Midgut of Beet Armyworm, Spodoptera exigua
Source: Int J Mol Sci. 2023 Feb 4;24(4):3076. doi: 10.3390/ijms24043076 (PMC9961250; doi:10.3390/ijms24043076)
Supplement: Supplementary file 1 [file ijms-24-03076-s001.zip › ijms-2113458-supplementary.pdf]

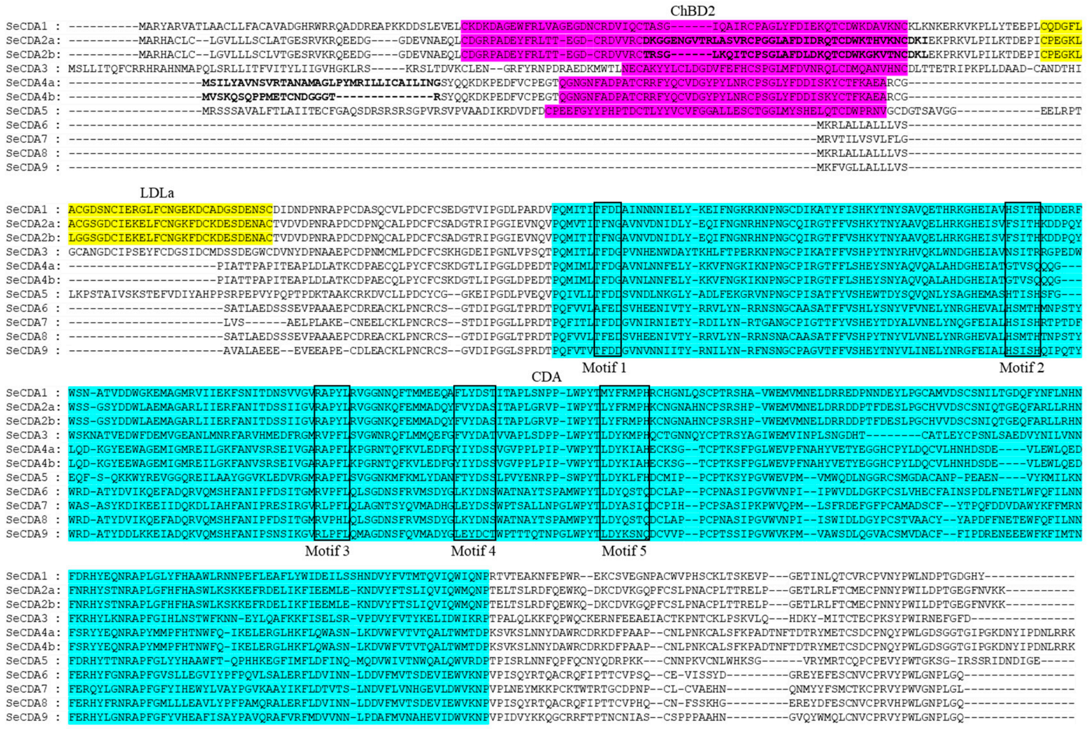

Figure S1. Amino acid sequence alignment of chitin deacetylases from *S. exigua*. The chitin binding domain is represented by purple background, the low-density lipoprotein receptor binding domain is represented by yellow background, and the blue background is the chitin deacetylation catalytic domain. Alternate mosaic areas of CDA2a, CDA2b and CDA4a, CDA4b are shown in bold. The black boxes indicate five conserved motifs in the CDA domain. Two forward slashes (//) indicate other amino acids not shown (SeCDA5 is 1020 amino acids).

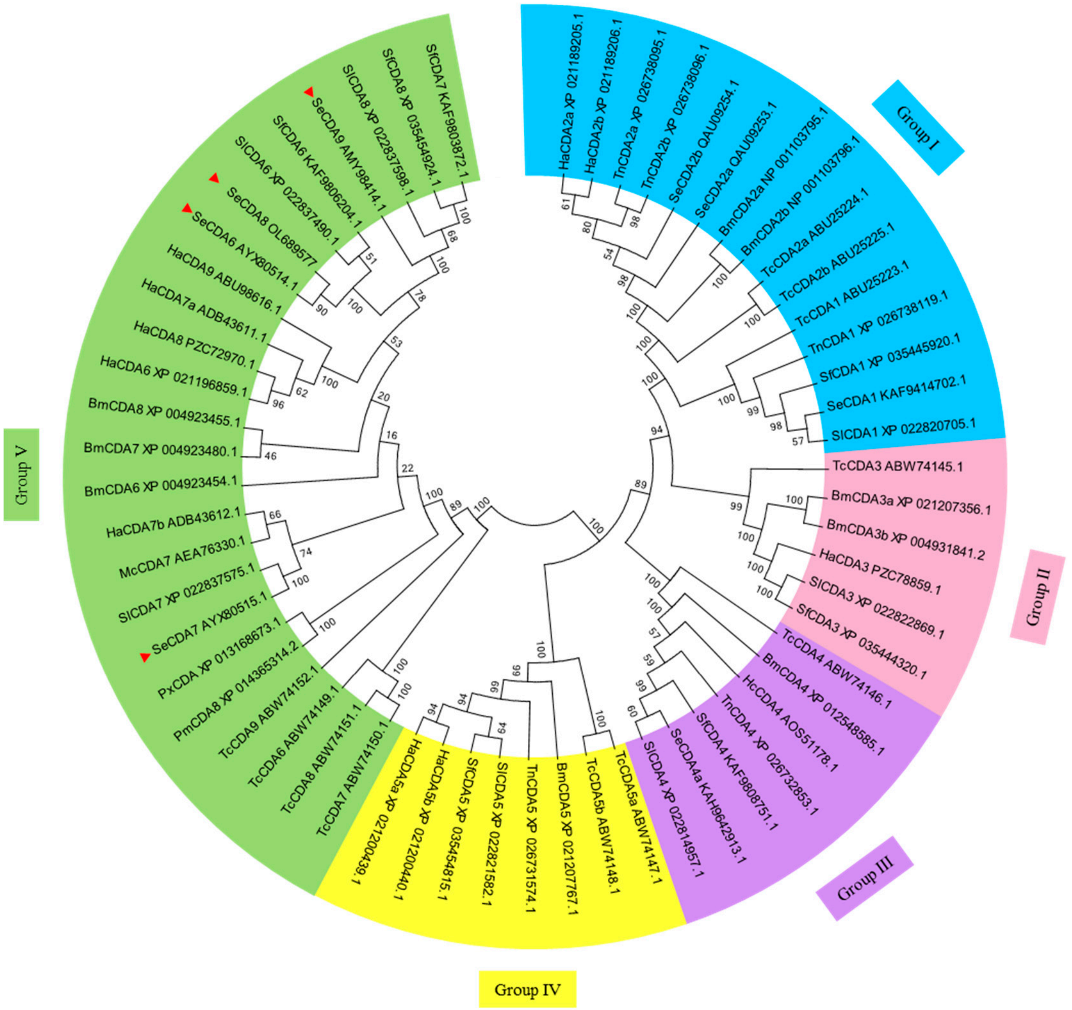

Figure S2. Phylogenetic tree of CDAs amino acid residue sequence of *S. exigua* and other insects by Minimum-Evolution. SeCDAs of *S. exigua* are labeled with a red solid triangle. The origin of CDAs species: Bm: *Bombyx mori*; Ha: *Helicoverpa armigera*; Hc: *Hyphantria cunea*; Mc: *Mamestra configurata*; Pm: *Papilio machaon*; Px: *Papilio Xuthus*; Sf: *Spodoptera frugiperda*; Sl: *Spodoptera litura*; Tc: *Tribolium castaneum*; Tn: *Trichoplusia ni*.

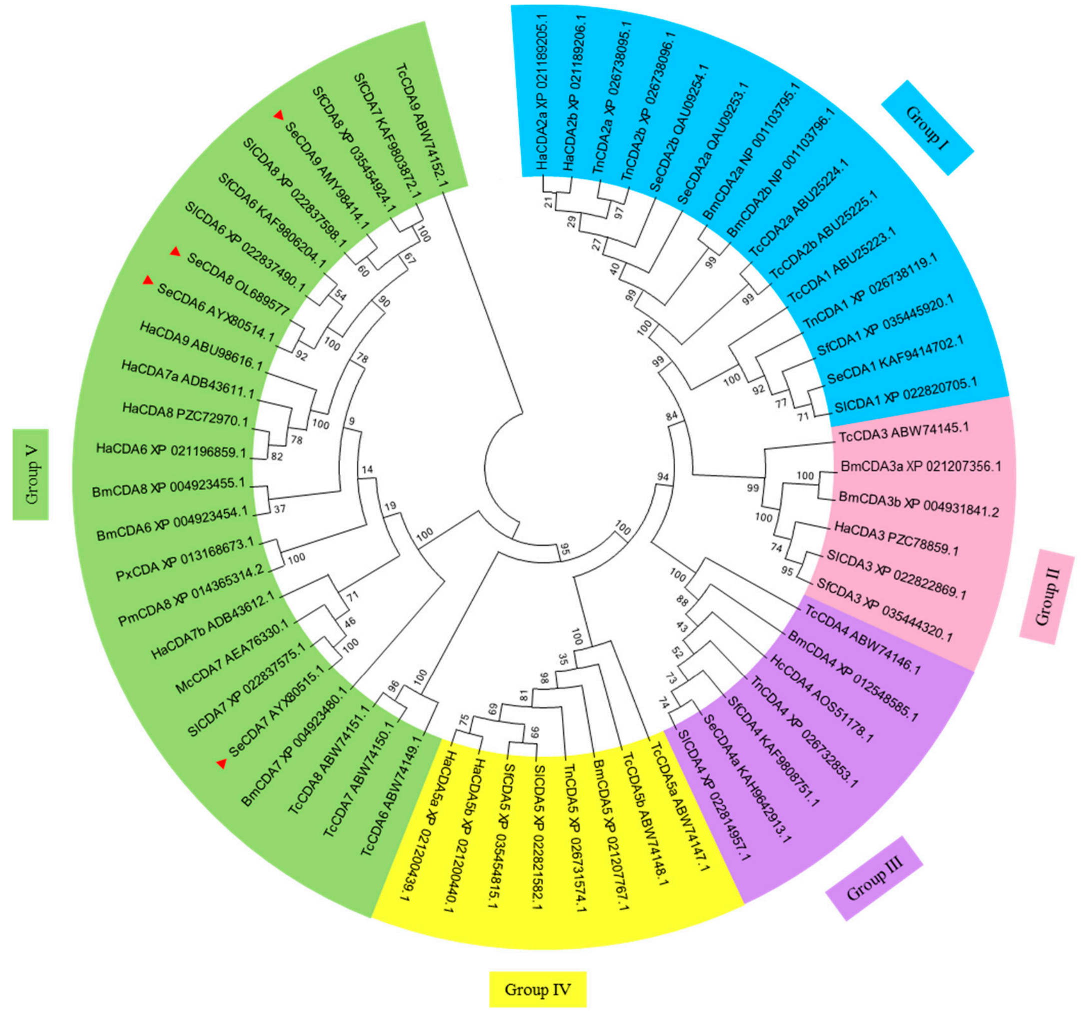

Figure S3. Phylogenetic tree of CDAs amino acid residue sequence of *S. exigua* and other insects by Maximum Likelihood. SeCDAs of *S. exigua* are labeled with a red solid triangle. The origin of CDAs species: Bm: *Bombyx mori*; Ha: *Helicoverpa armigera*; Hc: *Hyphantria cunea*; Mc: *Mamestra configurata*; Pm: *Papilio machaon*; Px: *Papilio Xuthus*; Sf: *Spodoptera frugiperda*; Sl: *Spodoptera litura*; Tc: *Tribolium castaneum*; Tn: *Trichoplusia ni*.

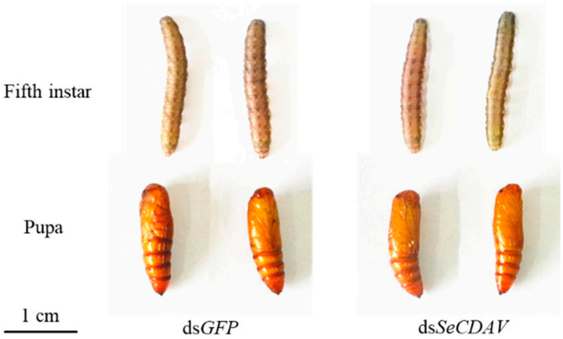

Figure S4. The phenotypes of larvae after dsRNA-*SeCDAV*/*GFP* injection. Scale bars were 1 cm.

All methods and data were confirmed to follow the Minimum Information for Publication of Quantitative Real-Time PCR Experiments (MIQE) guidelines. For the ΔΔCt calculation to be valid, the amplification efficiencies of the target and reference must be approximately equal.

The results of an experiment where a cDNA preparation was diluted over a 100-fold range shows in Supplementary Figure S5.

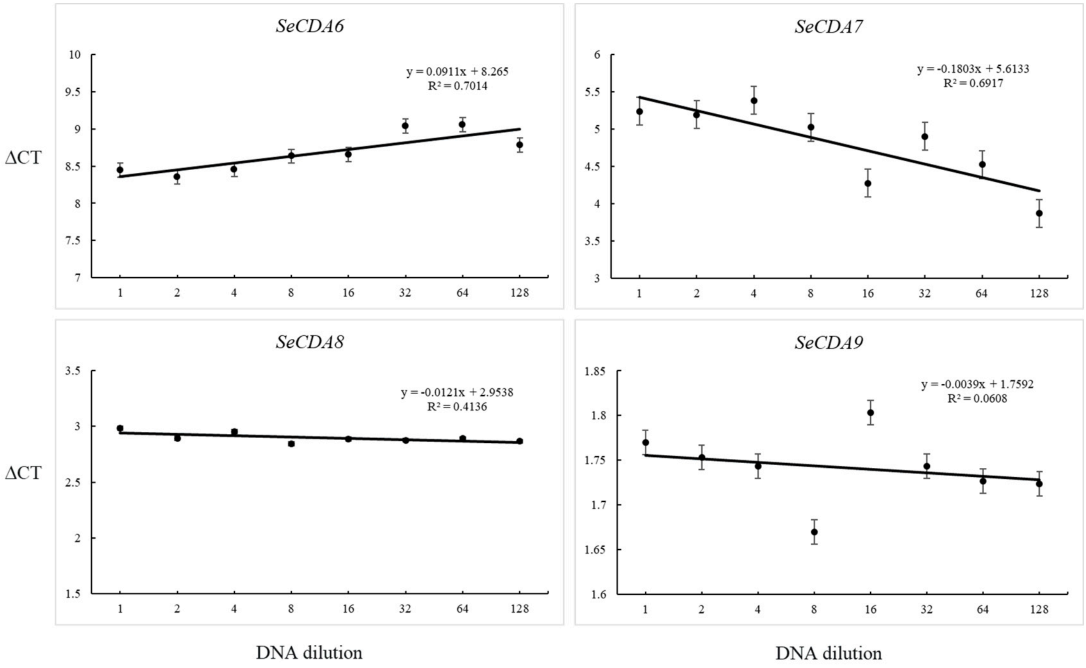

Figure S5. Validation of the $2^{-\Delta\Delta CT}$ method: Amplification of cDNA synthesized from different amounts of RNA. The efficiency of amplification of the target gene (*SeCDAs*) and internal control (*β-actin*) was examined using real-time PCR. Using reverse transcriptase, cDNA was synthesized from 1 ug total RNA isolated from *S. exigua*. Serial dilutions of cDNA were amplified by real-time PCR using gene-specific primers. The most concentrated sample contained cDNA derived from 7.8125 ng of total RNA. The ΔC_T (C_T. *SeCDA* - C_T. *β-actin*) was calculated for each cDNA dilution. The data were fit using least-squares linear regression analysis (N = 3).
